# Study protocol: a double blind placebo controlled trial examining the effect of domperidone on the composition of breast milk [NCT00308334]

**DOI:** 10.1186/1471-2393-6-17

**Published:** 2006-05-23

**Authors:** Marsha L Campbell-Yeo, Alexander C Allen, K S Joseph, Joyce M Ledwidge, Victoria M Allen, Kent C Dooley

**Affiliations:** 1 Neonatal Intensive Care Unit, IWK Health Centre and Dalhousie University, Halifax, Nova Scotia, Canada; 2 Doctoral Candidate, McGill University, Quebec, Canada; 3 the Perinatal Epidemiology Research Unit, Departments of Obstetrics & Gynecology and Pediatrics, IWK Health Centre and Dalhousie University, Halifax, Nova Scotia, Canada; 4 Laboratory Services, IWK Health Centre and Dalhousie University, Halifax, Nova Scotia, Canada

## Abstract

**Background:**

Domperidone, a drug that enhances upper gastric motility, is an anti-dopaminergic medication that also elevates prolactin levels. It has been shown to safely increase the milk supply of lactating women. To date, researchers have analyzed the effects of domperidone on lactating woman with respect to the quantity of their milk production, adverse effects, and drug levels in the breast milk. However, the effect of domperidone on the macronutrient composition of breast milk has not been studied and current guidelines for fortification of human milk for premature infants do not distinguish between those women using or those not using domperidone. The purpose of this study is to evaluate the effect of domperidone (given to lactating mothers of very preterm infants) on the macronutrient composition of breast milk.

**Methods/Design:**

Mothers of infants delivered at less than 31 weeks gestation, who are at least 3 weeks postpartum, and experiencing lactational failure despite non-pharmacological interventions, will be randomized to receive domperidone (10 mg three times daily) or placebo for a 14-day period.

Breast milk samples will be obtained the day prior to beginning treatment and on days 4, 7 and 14. The macronutrient (protein, fat, carbohydrate and energy) and macromineral content (calcium, phosphorus and sodium) will be analyzed and compared between the two groups. Additional outcome measures will include milk volumes, serum prolactin levels (measured on days 0, 4, and 10), daily infant weights and breastfeeding rates at 2 weeks post study completion and at discharge. Forty-four participants will be recruited into the study. Analysis will be carried out using the intention to treat approach.

**Discussion:**

If domperidone causes significant changes to the nutrient content of breast milk, an alteration in feeding practices for preterm infants may need to be made in order to optimize growth, nutrition and neurodevelopment outcomes.

## Background

During the past two decades, significant advances in medical technology have contributed to the increased survival of preterm, very low birth weight infants. As mortality rates have declined, the focus has shifted to decreasing morbidity and length of hospital stay for these high-risk infants. Nutritional care is one area with great potential for improvement. There continue to be many unanswered questions as to what constitutes optimal nutrient intake for the extremely preterm neonate. Although the current literature states that fortified human breast milk is most desirable [1-6], the optimal macronutrient composition of prepared fortifiers continues to be studied [7]. Also, the shift from formula feeding of preterm infants to human breast milk has caused an increase in mothers of preterm infants providing expressed breast to their babies over prolonged periods.

Many mothers of very preterm infants experience difficulty maintaining milk production over several months, and hence medications such as domperidone are often recommended to help augment milk supply. Such medications increase maternal prolactin levels and have been shown to significantly increase the quantity of breast milk a lactating woman produces [8-18]. Higher prolactin levels have been linked to accelerated decline in protein levels in term breast milk [19,21]. However, there is a paucity of information in the literature, on the effect of domperidone on the macronutrient content of breast milk. Although pharmacological augmentation of breast milk volume is valuable, carefully conducted quantitative studies are required to assess the effect of such medication on human breast milk composition including protein, fat and other constituents. We, therefore, designed a study to determine the effect of domperidone on the macronutrient (protein, fat, and energy) and macromineral (calcium, phosphorus, and sodium) content of breast milk among mothers of very preterm infants.

## Summary of literature review

### a. Advantages of human breast milk

Breast milk is generally considered to be the optimal form of nutrition for all infants regardless of gestational age [1,2]. Preterm infants have better outcomes if they receive human breast milk during the first months after birth. Human breast milk provides both nutritive and nonnutritive benefits to preterm infants. Nutritive benefits include a whey predominant protein source, which is more easily digested and promotes more rapid gastric emptying. Human milk is also a source of the very long chain fatty acids, which are important components of brain and red cell membranes. Nonnutritive advantages include psychological benefits of maternal-infant bonding, improved gastrointestinal outcomes, reduced risk of atopic eczema, and fewer systemic infections during initial hospitalization, [3-7,24-28] as well as, improved psychomotor development [26-29].

### b. Expressed breast milk

Increasingly, mothers of premature babies being cared for in neonatal intensive care units are asked to provide expressed human breast milk for their babies. Most mothers express a strong desire to provide breast milk for their infant and verbalize positive feelings about being able to do something to help their ill baby. However, many mothers of very preterm infants find it difficult to provide adequate amounts of expressed breast milk to meet their baby's needs over prolonged periods of time. Stress related to the hospitalization, concerns related to the increased risk of mortality and morbidity, separation, and lack of infant suckling may contribute to decreased milk production [33]. Decreased milk production among mothers of very preterm infants expressing breast milk can occur as early as two weeks postpartum, although such problems typically peak between four and six weeks postpartum [8,33].

### c. Physiology of lactation

The initiation of lactation (lactogenesis) is a natural process that is based on a complex interaction of hormones. Changes in estrogen and progesterone during pregnancy alter the morphologic structure of breast tissue. Increasing levels of the hormone prolactin is key in the initiation and maintenance of lactation [34,35] and may play a role in the control of electrolyte composition [20,21] and alteration in amino acid concentration [19] of mature human breast milk. A significant increase in lactogenesis occurs following delivery with the concurrent decline in estrogen and progesterone. In addition, the tactile stimulus by the suckling baby causes a release of prolactin from the pituitary, which stimulates milk flow. This suckling induced prolactin release is a reflex related to hypothalamic serotoninergic neuron activation, accompanied by a concomitant increase of dopamine turn over [34,35]. Babies delivered at less than 32 weeks gestation are not able to effectively coordinate sucking, swallowing and breathing [36]. The lack of effective suckling that is associated with the delivery of a preterm baby as well as maternal stress, infection, or fatigue can lead to difficulties maintaining lactation [8,33].

### d. Non-pharmacological interventions to enhance human breast milk production

Non pharmacological measures such as added emotional support, kangaroo care/skin to skin, compressing and massage, relaxation techniques, expressing breast milk at the baby's bedside, increasing pumping times, and alterations in mechanical expression contribute to a variable level of success in augmenting milk production [9,37]. For those mothers whose milk production is refractory to non-pharmacological interventions, and who verbalize a continued desire to provide expressed breast milk, the use of pharmacological intervention may be recommended.

### e. Pharmacological interventions

The administration of an anti-dopaminergic drug, such as metoclopramide or domperidone has been shown to yield positive results. Endogenous dopamine has a physiological inhibitory role in the control of prolactin release. Therefore, the administration of a dopamine inhibitor increases plasma prolactin levels and induces lactogenesis [9]. Metoclopramide, a central dopamine antagonist, has been most widely studied and proven to be highly efficacious in augmenting milk supply [8,10-14]. However, reports indicate that it crosses the blood brain barrier, and is secreted in significant amounts in the breast milk [12]. As well, animal studies have reported dopamine mediated responses in the offspring of nursing rats [38].

### f. Why choose domperidone?

Both metaclopramide and domperidone are anti-dopaminergic drugs. Both induce lactation by inhibiting endogenous dopamine and thereby increasing plasma prolactin levels. Domperidone is a newly developed anti-emetic with no structural relationship to the phenothiazine anti-emetics and metoclopramide; it is structurally related to the neuroleptic drug droperidol. Domperidone is a dopamine antagonist in vitro; it binds strongly to the dopamine (DA) receptors of the striatal area [40]. It is felt the domperidone inhibits dopamine receptors at either the level of the anterior pituitary median eminence or at the tuberoinfundibular systems, both of which are outside the blood brain barrier. This differs significantly from metoclopramide, which inhibits dopamine (DA) centrally [9]. Domperidone is less lipid soluble, has a higher molecular weight and has lower protein binding as compared with metoclopramide [84]. These characteristics may also prevent domperidone from crossing the blood brain barrier, thus causing less extra pyramidal effects that are more commonly reported with the use of metoclopramide.

Thus, domperidone, a peripheral dopamine antagonist, may be safer than metoclopramide [39]. Drug levels of domperidone in breast milk are also significantly lower than metoclopramide (p < 0.05) [15,16]. Studies have shown that breast milk samples taken from women receiving either a 10 mg dose of domperidone or a 10 mg dose of metoclopramide contained 10.3 ng/ml of domperidone and 68.5 ng/ml of metoclopramide (p < 0.05) [16]. Domperidone has been shown to significantly increase milk production without evidence of adverse effects in the infant [15-18]. Domperidone has been shown to raise the serum prolactin level in non-lactating women from 8.1 to 110.9 ng/ml after one 20 mg dose [40]. In a double-blind placebo controlled trial, 32 mothers of full term infants experiencing failure of lactogenesis were randomized to receive either domperidone (10 mg TID) or placebo. Prolactin levels were reported as significantly higher after the 2^nd ^day of treatment in the domperidone group (p < 0.01). The mean daily milk yield was also increased significantly in the domperidone group (P < 0.01), with a corresponding higher infant weight gain [18]. In a small double blind, randomized controlled trial (n = 16), a 7 day course of domperidone (10 mg TID) given to mothers of preterm infants experiencing insufficient milk production resulted in a significantly increased milk supply (P < 0.05), low levels of drug detected in breast milk (< 0.2 mcg/kg/d) and significantly higher maternal prolactin level when compared to the placebo group [17]. No infant or maternal side effects were reported in the above trials. Both the Canadian and American Academy of Pediatrics have approved the use of domperidone for breast-feeding mothers [41].

### g. Fortification of human breast milk

Despite the numerous advantages of human breast milk, the exclusive feeding of human breast milk to premature infants has been associated with lower rates of growth, nutritional deficits, and prolonged hospital stays [42-51]. Commercial formulas for preterm infants have been designed to meet the nutritional needs of such infants but cannot provide the infant with the advantages of human milk [3-7,22-32]. Therefore, it is generally recommended that breast milk feeds for preterm infants be fortified with a commercially available human milk fortifier (HMF) [2,7,52,53]. The inadequate intake of protein, energy, calcium, and phosphorus related to unfortified breast milk has been cited as the main reason for the need for fortifiers [42-50,55-58]. Infant fortifiers provide supplemental amounts of calories, protein, vitamins, minerals and trace elements to human breast milk in order to best meet the nutritional needs of the preterm infants.

Preterm infants are born with low skeletal stores of calcium and phosphate. They have increased requirements for these minerals in order to ensure adequate postnatal skeletal growth. Poor radiological bone mineralization, rickets and fractures have been described in premature infants receiving breast alone [55,56,58]. Supplementation of calcium and phosphorous has been shown to normalize biochemical indices, serum calcium, and serum phosphorus, as well as normal urinary excretion of calcium and phosphorous [59-61]. Serum sodium levels also normalize following supplementation [57].

Protein and energy supplementation has been associated with improved rates of weight gain and better indices of protein nutritional status [49]. The Cochrane review on protein supplementation states that protein supplementation of preterm mothers milk increases short-term weight gain: weighted mean difference 3.6 g/kg/day, 95% CI 2.4 to 4.8 g/kg/day, linear growth 0.28 cm/week, 95% CI 0.18 to 0.38 cm/week, and head growth 0.15 cm/week, 95% CI 0.06 to 0.23 cm/week. Blood urea levels also increase with protein supplementation 1.0 mmol/l, 95% CI 0.8 to 2.1 mmol/l [62]. Current supplemental feeding practices are calculated based on anticipated macronutrient and electrolyte composition of preterm mother's milk that is not being augmented by medication.

### h. Breast milk composition

Human breast milk composition is widely variable both within and between breast-feeding mothers [63]. Variation in macronutrient composition can occur at various feeding times throughout the day or night and also at the beginning and end of a feed [64]. These variations have been reported to be greater in mothers of preterm infants providing expressed breast milk. Measuring samples taken from pooled 24-hour collections may limit the degree of variability reported. Variation in the composition of human breast milk has been associated with the diagnosis of either sporadic or chronic mastitis. The sodium content of the breast milk is the constituent most profoundly affected [66].

The composition of preterm mother's milk also differs from that of term mother's milk [67-69]. Although protein levels are initially higher in preterm breast milk, the natural temporal decline of protein content creates a nutritional deficit when compared to the needs of the preterm infant [5]. Studies show that there is a decline in protein content following delivery, while fat, lactose and energy content increases. These trends are more pronounced during the first 3 weeks and are greatest in the preterm group [64]. In a more recent study, random spot samples of breast milk from mothers of premature infants were analyzed to estimate macronutrient variability. Although fat content was highly variable within and between individuals, it remained fairly constant throughout lactation. Protein was less variable but was found to decrease significantly throughout the first four weeks of lactation (r = -0.45, p < 0.001), then continued to fall at a slower rate of decline over time and with increased volumes of breast milk [70]. Several studies have reported similar findings [67,70-74].

Maas [74] studied the effects of gestational age (GA), postnatal age (PNA), and post-menstrual (PMA = GA+PNA) on the macronutrient composition of very preterm mothers milk. Total nitrogen, fat, lactose and carbohydrate concentrations, energy density and 24-hour volume were determined from 282 (24 hour) samples collected (day 7–55) from seventy mothers of premature infants (25–29 weeks gestation) [74]. The major findings of the study were that developmental changes in milk composition are largely determined by PNA, minimally by GA and not at all by PMA. Milk volume (24 h) itself did not show a dependence on GA, PNA or PMA. When volume was used as a time varying covariate in the analysis, total nitrogen content decreased while lactose and carbohydrate content increased when 24-hour milk volume increased. These findings were in accordance with the literature [70,71,73].

There have been two studies examining the effect of metoclopramide on macronutrient content and sodium content of term mother's milk. When term breast milk samples from primiparous women taking metoclopramide were compared to samples from a similar group taking a placebo, the expected decline in protein content occurred at an accelerated rate in the treatment group [19]. Sodium concentration in term human breast milk was compared in a smaller study using a similar design to determine the effect of metoclopramide. No significant differences were detected between groups. The small sample size (n = 22) and lack of data regarding calcium and phosphorus levels warrant further investigation in relation to preterm milk composition [21]. Since domperidone differs in structure and mechanism of action from metoclopramide and since studies evaluating metoclopramide's effect on breast milk composition have been limited to term mother's milk, our study is both warranted and necessary to ensure optimal infant nutrition.

## Hypothesis

The macronutrient composition (protein, fat and calories) of human breast milk will be significantly altered by the administration of domperidone.

## Methods/Design

### a. Objectives

The intent of this study is to evaluate the effect of domperidone on the macronutrient composition of breast milk among mothers of preterm infants. Potential changes in protein, fat, lactose, energy, calcium, phosphorus and sodium content in expressed breast milk will be assessed. Secondary objectives include assessment of the effect of domperidone on breast milk volume, prolactin level, daily infant weight, and breastfeeding rates 2 weeks after treatment completion and at discharge.

### b. Study design

We propose to carry out a randomized double-blind placebo-controlled study comparing the effect of domperidone use on the macronutrient composition of preterm mother's milk.

### c. Study population

The source population consists of mothers of preterm infants delivered at less than 31 weeks gestational age admitted to the Neonatal Intensive Care Unit at the IWK Health Centre who are providing expressed breast milk to their infants and who experience lactational failure three weeks after delivery.

### d. Inclusion criteria

i. Mother of an infant born less than 31 weeks gestation

ii. Women mechanically expressing breast milk using a double collecting system

iii. Experiencing lactation failure indicated by at least one of the following:

A decreasing milk supply (by greater than 30 percent from peak volume based on maternal account)

An inability to provide adequate breast milk to meet the daily nutritional intake of their infant

iv. Women who have had little or no improvement in milk production following education/counseling with a lactation consultant/Neonatal Intensive Care nurse with respect to non-pharmacological techniques.

v. Postpartum period equal to or greater than three weeks.

### e. Selection criteria for study subjects

Women who meet the inclusion criteria will be approached by the study investigator/nurse, informed of the study. Signed written informed consent will be obtained.

### f. Exclusion criteria

i. Participants receiving any medication known to alter the effect of domperidone (e.g. cimetidine, ranitidine, famotidine, and nizatidine) or medication that interacts with domperidone (e.g. haloperidol, lithium).

ii. Experiencing mastitis

iii. Having a chronic or debilitating illness.

iv. Previous breast surgery

v. Having a known lactose intolerance

## Allocation of participants to the trial groups – see figure 1

**Figure 1:**
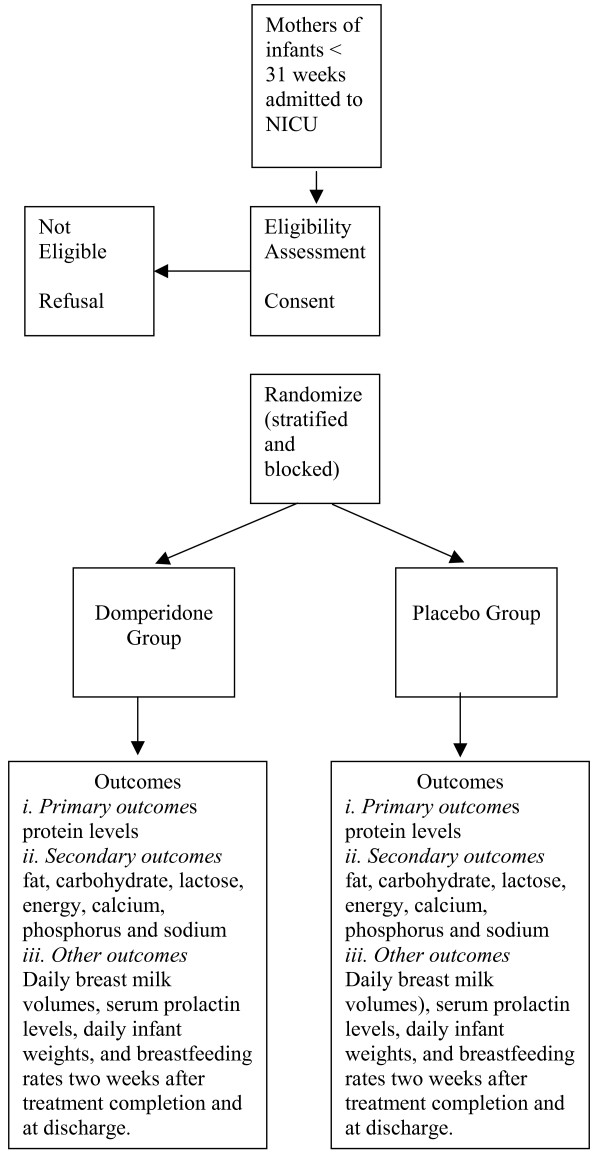
Allocation of Participants to the Trial Groups.

### Randomization

Eligible and consenting woman will be randomized in blocks of four. Pharmacy staff will randomly (using a computer based code) assign mothers to receive either domperidone, 10 mg orally three times daily [17,38] or placebo for a two week period. Mothers of infants >3 but < 4 weeks postpartum will be randomized separately from those ≥ 4 weeks postpartum in order to ensure identical proportions within the domperidone and placebo groups (i.e. randomizations will be stratified by postpartum duration at study entry). Participants will receive their 2- week drug or placebo supply following enrollment. They will be provided with instruction related to self-administration and asked to return the medication vial and any unused medication at the completion of the two-week period. The study nurse/investigators will be in close contact with the mothers and review instruction related to medication on study days 4, 7, and 14.

## What are the proposed methods of protecting against sources of bias?

The double blind study design in which participants, neonatal staff, and study personnel will be blinded to group enrollment will ensure that non-domperidone related intervention effects and information collection is similar between the drug and placebo groups. In order to have similar presentation and appearance of domperidone and placebo, domperidone tablets will be crushed and mixed with lactose. The resulting powder will be placed in clear capsules. Plain lactose powder will also be placed in clear capsules to act as the placebo. Participants will receive a two-week supply of either domperidone or placebo following randomization at no cost to them. Measured prolactin levels will not be made available until the completion of the study period.

## What is the proposed duration of treatment?

The treatment period begins following randomization. It consists of day 0 on which baseline measurements will be taken and recorded, and a 14-day treatment period during which domperidone (10 mg × 3/daily) or placebo (3/daily) is administered.

## What is the proposed frequency and duration of follow-up?

Breast milk samples will be obtained on day 0, 4, 7, and 14. Serum prolactin levels will be collected on days 0, 4, and 14. Infant weights will be measured daily. Each participant will be asked questions regarding breast-feeding two weeks following discontinuation of the treatment and at discharge.

## What are the proposed outcome measures?

### a. Primary outcome

The protein levels from breast milk, collected on days 0, 4, 7, and 14 will be compared, between the two groups (taking into consideration the expected rate of decline associated with increasing postpartum days).

### b. Secondary outcomes

The fat, carbohydrate, lactose, energy, calcium, phosphorus and sodium content of breast milk, collected on days 0, 4, 7, and 14, will be compared between domperidone and placebo groups.

### c. Other outcomes

Daily breast milk volumes (if the infant breast feeds, volume will be estimated using pre and post feeding weights), serum prolactin levels, daily infant weights, and breastfeeding rates two weeks after treatment completion and at discharge.

## Sample size

Sample size was calculated using a 2-sided test, an alpha error of 0.05 and a power of 80 percent. Based on the medical literature and prior clinical understanding, we designed the study to detect as significant a 20 percent or greater change in protein content (in the domperidone group relative to the placebo group). We will also have sufficient power to detect a 20 percent or greater change in fat content and a 10 percent or greater change in energy content, if such differences are in fact caused by domperidone. Means and standard deviation values for protein, fat and energy content used in the sample size calculation were obtained from a recent, authoritative publication on the breast milk content of preterm human milk [74]. Based on this calculation our study will recruit 44 participants, 22 assigned to the domperidone group and 22 to the placebo group.

## What is the planned recruitment rate?

In the year 2000, 58 surviving infants less than 31 weeks gestation were admitted to the Neonatal Intensive Care Unit. Of these 20–25 percent were multiple births. Breastfeeding initiation rates are 80–85 percent in our nursery. Due to the lack of information regarding the incidence of lactation failure in this population, a convenience (2001) survey of mothers of preterm infants providing breast milk for their babies was carried out. Fifty-five percent of these mothers had experienced lactation failure, several of whom had been prescribed domperidone (domperidone is not prescribed routinely in this centre). Therefore, 20–23 women a year will be eligible for the study. Recruitment rates for research studies in our NICU range from 60–100 percent. Based on previous studies done in this setting where risk is minimal and side effects are rare, we estimate a greater than 90 percent recruitment rate.

## Will compliance be a concern?

Since side effects are rare, we do not anticipate problems with compliance. The majority of mothers will be visiting their baby on a regular basis, hence providing milk and blood samples should not be a great inconvenience. Participants will receive their 2-week supply of domperidone or placebo following enrollment. They will be provided with instruction related to self-administration and asked to return the medication vial and any unused medication at the completion of the two-week period. Pill counts will be done in pharmacy to ensure drug accountability and evaluate compliance. The study nurse/investigators will be in close contact with the mothers and review instruction related to medication on study days 4, 7 and 14.

Based on our past clinical observations, we anticipate that the majority of the participants enrolled will be mothers of infants delivered between 24–28 weeks gestational age. The infants will be 29–33 weeks corrected gestational age at the end of the study period. Although many of these infants will receive skin to skin care from their mothers (kangaroo care), few will be actively breast feeding. For those infants who are enrolled at a later gestational age (who will be closer to 35 weeks at the end of the study period) and able to begin breast-feeding, milk volumes will be calculated using pre and post feeding weights. Mothers will be asked to refrain from breast-feeding and to express breast milk on days 0, 4, 7 and 14 as per our usual practice to ensure that a 24-hour pooled breast milk sample will be obtained. Recruitment and compliance therefore will not be adversely affected.

## What is the likely rate of loss to follow Up?

The Neonatal Intensive Care Unit (NICU) is the tertiary level neonatal unit for all of Nova Scotia and for most of the Maritime Provinces. Most babies in the NICU stay until discharged from hospital. Approximately 20 percent return to local hospitals prior to discharge home. However, very few are transferred prior to 35 weeks corrected gestational age. Mothers of any infants transferred prior to discharge will be provided with a toll free telephone number and instructed to notify the principal investigator or study nurse of their discharge home. Answers to follow-up questions will be obtained by telephone by either the principal investigator or study nurse. In addition, most infants that meet inclusion criteria for this study (all Nova Scotia and Prince Edward Island babies born at less than 31 weeks) continue to be followed until three years of age as part of the IWK Perinatal Follow-up Program. This program loses two to three percent to follow-up. We therefore do not expect any significant losses to follow up.

## What is the proposed type and frequency of analysis?

### a. Method of breast milk collection

Throughout the study, breast milk will be expressed according to the standard practice of the nursery. Breast milk will collected using the Symphony reusable breast pump (Medela Canada). Each participant will be supplied with a double pump collecting system and sterile collection bottles. Each woman will be instructed to collect 24-hour samples of breast milk for the day prior to beginning treatment and then each day for the two-week period. A new container will be used for each pumping. Each participant will keep a diary to record the amount of milk pumped, the date and time as well as ensure that adhesive labels with this information also be placed on each of the containers. Daily milk volumes will be calculated. On days 0, 4, 7 and 14, two small samples (1 ml and 30 mls) of milk (from a pooled 24 hour collection) will be retained for analysis. The remainder of the milk will be available for the infant as usual.

### b. Chemical analysis

Breast milk samples (24 h – pooled) will be frozen (-70 C) and couriered in batches of eight for analysis of macronutrient content to the Prince Edward Island Food Technology Centre. Total nitrogen will be determined using the Kjeldahl method [75]; fat by the Roese-Gottlib method; ash and moisture by Forced Air Oven: and, energy, carbohydrate and lactose concentrations will be calculated [78].

Mineral content will be analyzed at the IWK Laboratory Department. Samples will be centrifuged and the aqueous component assayed [79]. Phosphorous content will be measured using the Ammonium molybdate method on the Ortho Vitros^®^) chemistry analyzer [80]. Calcium content will be determined using the Arseno (III) method on the Ortho Vitros^®^) chemistry analyzer [81]. Sodium content will be determined by ion selective electrode [82].

Mothers will also be asked to provide blood samples to determine prolactin concentrations prior to the initial dose, on day four and day 14. Serum prolactin levels will be collected at the IWK Health Centre outpatient tab and measured using Chemiluminescence immunoassay on the Vitros^®^) Eci analyzer [83]. Each participant will receive, requisitions for blood collection dated for the appropriate study days required. If a participant is unable to come to the IWK Health Centre, the principle investigator or study nurse will make alternate arrangements with her family doctor and local laboratory to ensure that the specimen is collected.

### c. Statistical analysis

Analysis and inference will be based on the intention-to-treat principle. Efforts will be made to ensure that follow up is complete for all subjects and that there are no missing values for any of the subjects for any variable. Blinding of group assignment will be retained until after the analysis is completed. Baseline characteristics of study subjects will be contrasted to ascertain that randomization has in fact produced comparable groups with respect to all variables that effect the composition of breast milk including gestational age at delivery and duration since delivery. The primary outcome of interest will be a potential decline in breast milk protein content over time within the domperidone group versus the placebo group. This analysis will compare the means in the two groups before and after treatment and contrast the mean difference between groups using 95 percent confidence intervals and a p value. The stratified nature of the randomization will be accounted for in the analysis. If differences are noted in baseline characteristics, inferences will be made based on observed and (linear regression) adjusted differences between groups. Analysis for fat, lactose, energy, calcium, phosphorus and sodium content in expressed breast milk will be done in an identical fashion as for protein by comparing the means in the two groups before and after treatment and contrasting the mean differences between groups using 95 percent confidence intervals and a p value. Prolactin levels will be compared between the two groups and used to determine the correlation between prolactin levels and volume of milk produced.

## Trial management

The study nurse will assume the role of study coordinator. She will be responsible for the day-to-day management of the trial. The principal investigator will have an active role in the trial and meet weekly with the study nurse.

## Significance of study

To date, researchers have analyzed the effects of domperidone on lactating woman with respect to the quantity of their milk production, adverse effects, and drug levels in the breast milk. Currently, guidelines for fortification of human milk for preterm infants do not distinguish between women using or not using domperidone. Domperidone causes an increase in maternal prolactin levels and has been shown to significantly increase human breast milk volumes. No study has examined the effect of domperidone use (to augment lactation) on the composition of breast milk. The purpose of this study is to compare the macronutrient and macro mineral composition of breast milk between mothers of infants delivered at less than 31 weeks gestation, receiving domperidone versus placebo. If significant differences in nutrient content occur between groups, alterations to current standards of feeding practices for premature infants may need to be made in order to optimize growth and nutrition. The results of this study will provide clinicians with valuable information to aid in guiding feeding practices for preterm infants.

## Potential risks to the safety of participants involved in the study

### a. Potential side effects of medication

Side effects associated with the use of oral domperidone are extremely uncommon. Side effects that have been reported include dry mouth, transient skin rash or itching, headache, thirst, abdominal cramps, diarrhea, drowsiness and nervousness. These side effects have been reported only occasionally (incidentally) and have been generally associated with high dosages of the medication. Symptoms are usually minor and all symptoms disappear following discontinuation of the medication [41,76].

Domperidone is highly metabolized by CYP3A4 in the liver, drug interactions related to its inhibition are expected to occur. Cimetidine, ranitidine, famotidine, and nizatidine may interfere with domperidone's bioavailability and should not be co-administered [76]. Medications such as haloperidol and lithium when taken at the same time as domperidone may cause exaggerated central nervous system symptoms. Therefore, subjects who satisfy eligibility criteria will not be invited to participate in this study if they are taking any of these medications [76].

Low levels of domperidone are excreted in human breast milk [36,38]. There are no reported cases of side effects in infants. As previously mentioned, the Canadian and American Academy of Pediatrics have approved its use in breastfeeding mothers [41].

### b. Confidentiality

Participant data collected during this trial will be kept confidential and locked in a secure office area. Study staff will have access to the data as well as participant's medical records as they pertain to this study. A letter will be sent to the participant's family doctor to inform him/her of their participation in the study. Published results will not contain any information that would identify individual participants. Study records will be stored in a locked area and will be kept for 10 years past the age of majority of the infant.

## Role of each investigator

MCY will be responsible for the progress and timely completion of the trial. MCY, ACA, VMA and JML will be responsible for responding to clinical queries, encouraging recruitment, protocol compliance and accurate and complete data collection. KCD will be responsible for laboratory related queries. VMA will be responsible for prescribing medication and for providing advice regarding participant's concerns or questions. ACA will be responsible for providing advice regarding neonatal issues. KSJ will be responsible for providing advice on methodological issues and assisting with interpreting statistical analysis. Study investigators will be obliged to participate in study committee meetings related to study progress and completion.

## Ethics

Informed consent will be obtained from the women prior to study entry. The IWK Health Centre Research Ethics Board has approved this study. Domperidone is considered safe for use in lactating women. Nevertheless, a careful watch will be kept on all study participates with regard to side effects of drug administration. Participants will be made aware of possible side effects and given contact numbers in the event they experience an adverse effect. The research nurse or a member of the research team will ask each participant if they are experiencing any possible side effects on day 4, 7, and 14 of the study when breast milk samples are collected. Infants will be monitored as per the Neonatal Intensive Care Unit standard of care and their clinical condition will be evaluated daily as part of medical rounds.

The current standard of care at the IWK Health Centre Neonatal Intensive Care Unit does not recommend pharmacological management for lactation failure. The use of domperidone is based on the individual management practices of each woman's physician. There will be no study restrictions regarding the continued use of domperidone following the two-week study period. Any decision to do so would occur based on discussions between the participant and her family doctor. A letter informing the participant and the family doctor which study arm the participant had been randomized to will be sent following completion of the study.

## Study timeline

The initial preparation time for this study will be two weeks. During that time, information will be given to the staff of the Neonatal Intensive Care Unit regarding study protocol. Approximately 60 infants less than 31 weeks gestation are admitted to IWK yearly. Of these 20–25 percent are multiples. Breast-feeding initiation rates are 80–85 percent in our nursery. If half of these women experience difficulty with breast milk supply, 20–23 women a year will be eligible for the study. Recruitment rates are expected to be greater than 90 percent. Subject accrual and data collection will extend over 24 months. It will take an additional 4 months to analyze the data and prepare a manuscript. The study results will be widely disseminated through conference proceedings and peer reviewed publication. The total duration of the study is expected to be 28 months.

## Discussion

The first participant was enrolled in October 2003. In June of 2004 a Food and Drug administration (FDA, U.S.A.) warning was issued regarding safety concerns related to domperidone and lactating women. Seven women had been enrolled at that time. The IWK Health Centre Research Ethics Board was informed of the FDA warning and enrollment was discontinued. A No Objection Letter regarding the trial (Control # 093770) was issued by Health Canada October 1, 2004 and Ethics Approval was reinstated on October 5, 2004. An addendum was made to the consent form outlining the concerns raised by the FDA.

The FDA warning against the use of domperidone by lactating women was based on case reports related to the increased risk of cardiac arrhythmia and sudden death in patients with malignant disease receiving high-dose intravenous domperidone therapy concurrently with chemotherapy and experiencing hypokalemia (serum potassium levels between 2.0 and 3.3 mmol/L). In its oral form, domperidone has been approved for use in Canada and worldwide as a motility agent with an excellent safety record. In a randomized trial, DaSilva et al determined that in women receiving 10 mg of domperidone 3 times daily to enhance lactation, the mean serum level on day 5 of therapy was 6.6 ng/mL [17]. The mean level of domperidone excreted in the breast milk of these women was 1.2 ng/mL. No adverse effects to either mother or infants were reported.

## Competing interests

The author(s) declare that they have no competing interests.

## Authors' contributions

All authors contributed to the development of the protocol, and read and approved the final manuscript.

## Pre-publication history

The pre-publication history for this paper can be accessed here:


